# Tanycytes As Regulators of Seasonal Cycles in Neuroendocrine Function

**DOI:** 10.3389/fneur.2017.00079

**Published:** 2017-03-10

**Authors:** Jo E. Lewis, Francis J. P. Ebling

**Affiliations:** ^1^School of Life Sciences, University of Nottingham Medical School, Queen’s Medical Centre, Nottingham, UK

**Keywords:** tanycyte, season, photoperiod, neuroendocrinology, stem cells

## Abstract

Annual cycles of physiology and behavior are highly prevalent in organisms inhabiting temperate and polar regions. Examples in mammals include changes in appetite and body fat composition, hibernation and torpor, growth of antlers, pelage and horns, and seasonal reproduction. The timing of these seasonal cycles reflects an interaction of changing environmental signals, such as daylength, and intrinsic rhythmic processes: circannual clocks. As neuroendocrine signals underlie these rhythmic processes, the focus of most mechanistic studies has been on neuronal systems in the hypothalamus. Recent studies also implicate the pituitary stalk (*pars tuberalis*) and hypothalamic tanycytes as key pathways in seasonal timing. The *pars tuberalis* expresses a high density of melatonin receptors, so is highly responsive to changes in the nocturnal secretion of melatonin from the pineal gland as photoperiod changes across the year. The *pars tuberalis* in turn regulates tanycyte function in the adjacent hypothalamus via paracrine signals. Tanycytes are radial glial cells that persist into adulthood and function as a stem cell niche. Their cell soma are embedded in the ependymal lining of the third ventricle, and they also send elaborate projections through the arcuate nucleus, many of which terminate on capillaries in the median eminence. This anatomy underlies their function as sensors of nutrients in the circulation, and as regulators of transport of hormones and metabolites into the hypothalamus. *In situ* hybridization studies reveal robust seasonal changes in gene expression in tanycytes, for example, those controlling transport and metabolism of thyroid hormone and retinoic acid. These hormonal signals play a key role in the initial development of the brain, and experimental manipulation of thyroid hormone availability in the adult hypothalamus can accelerate or block seasonal cyclicity in sheep and Siberian hamsters. We hypothesize that seasonal rhythms depends upon reuse of developmental mechanisms in the adult hypothalamus and that tanycytes are key orchestrators of these processes.

## Introduction

**Figure 1:**
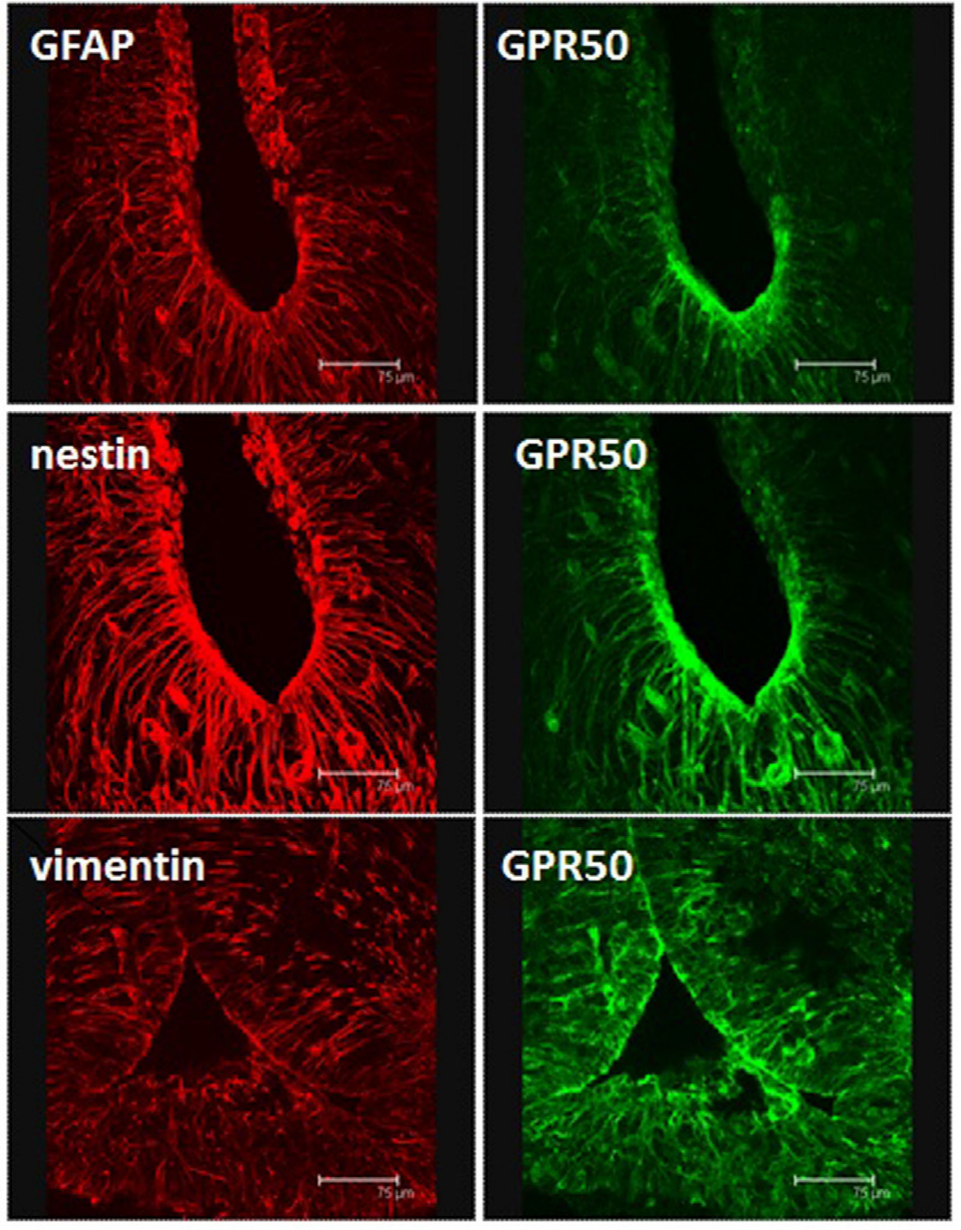
**Immunohistochemical identification of tanycytes in coronal sections through the mediobasal hypothalamus of a Siberian hamster**. Polyclonal rabbit antisera detect glial fibrillary acidic protein, or the intermediate filaments nestin or vimentin. Sections are also stained with a goat polyclonal directed against the melatonin-related receptor GPR50. Scale bars = 75 μm. Image from Fowler and Ebling, University of Nottingham.

**Figure 2:**
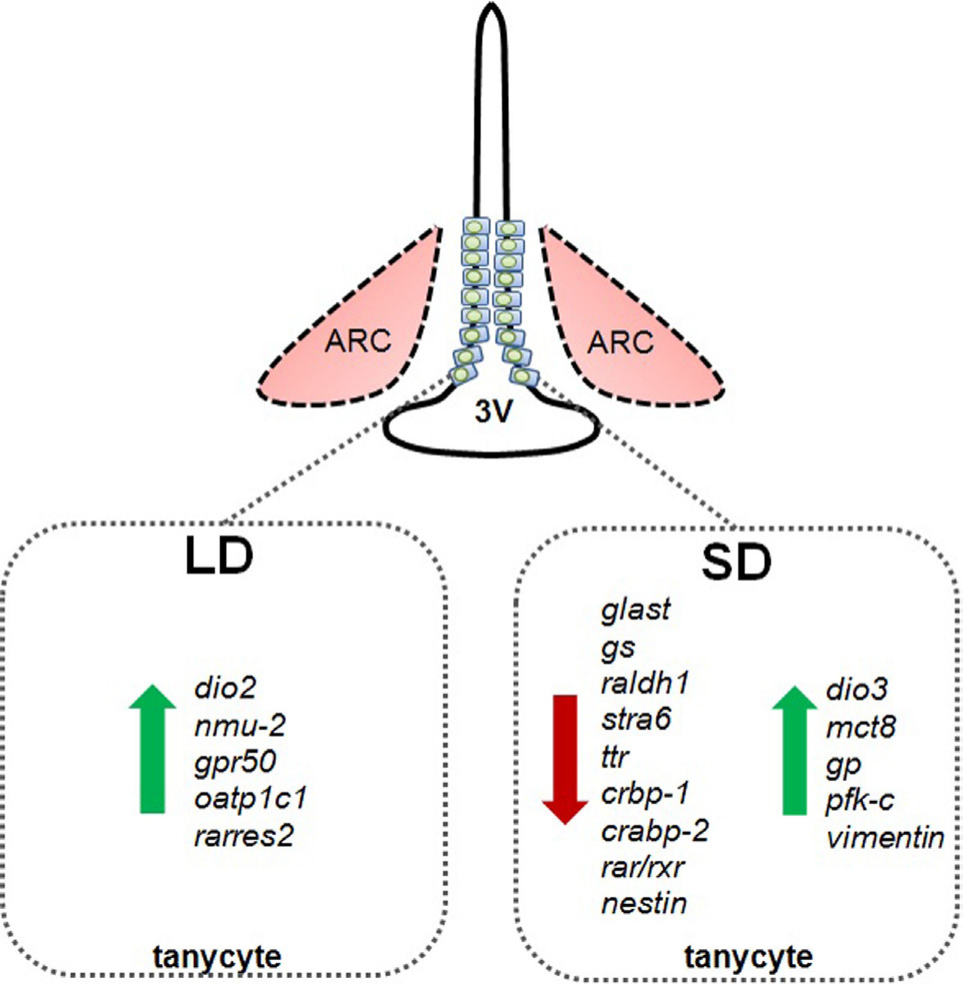
**Schematic summary of photoperiod-induced changes in gene expression in tanycytes in Siberian hamsters exposed to long summer photoperiods (LD) or short winter photoperiods (SD)**. *dio2*, deiodinase 2; *nmu-2*, neuromedin 2; *gpr50*, G-protein-coupled receptor 50 (=melatonin-related receptor); *oatp1c1*, organic anion transporter 1C1; *rarres2*, chemerin; *glast*, glutamate transporter; *gs*, glutamine synthetase; *raldh1*, retinaldehyde dehydrogenase; *stra6*, retinol transport protein stimulated by retinoic acid gene 6 homolog; *ttr*, transthyretin; *crbp-1*, cellular retinol binding protein; *crabp-2*, cellular retinoic acid binding protein-2; *rar/rxr*, retinoic acid and rexinoid receptors; *nestin*, type VI intermediate filament nestin; *dio3*, deiodinase 3; *mct8*, monocarboxylate transporter 8; *vimentin*, type III intermediate filament vimentin; *gp*, glycogen phosphorylase; *pfk-c*, phophofructokinase C.

Investigation of the central mechanisms underlying seasonal cycles in energy balance has provided new insights into the fundamental control systems of appetite and energy expenditure in the brain. Homeostatic mechanisms governing the short-term control of energy balance, for example, the timing of meals and the response to acute fasting, have been extensively studied in laboratory animal models. This body of work has given us great insight into the autonomic and endocrine signals emanating from the gastrointestinal tract and white adipose tissue that communicate to integrative centers of the hypothalamus and brainstem (1). However, the evidence that changes in homeostatic gene expression underlie long-term season cycles in energy balance is very limited (2, 3). In seasonal mammals, rheostatic mechanisms that govern the long-term control of energy balance reflect a higher order set of processes controlling the neuroendocrine system (4). A key element of this rheostatic system comprises hypothalamic tanycytes (Figure 1). These are radial glial cells whose cell soma in embedded in the ependymal lining of the third ventricle (Figure 1). They possess elaborate projections that communicate with hypothalamic nuclei implicated in energy balance (5). Subtypes of tanycyte have been identified on the basis of their location and their proximity to hypothalamic nuclei: α1 and α2 tanycytes appose the dorsomedial and ventromedial nuclei, whereas β1 and β2 tanycytes border the arcuate nucleus and median eminence. Interestingly, β2 tanycytes differ from the other subtypes as they have direct access to circulating plasma (6). These tanycytes in the ventral region of the third ventricle are uniquely fenestrated and selectively permeable, allowing passive and active transport of molecules from the circulating blood supply in the median eminence into the cerebroventricular fluid in the third ventricle (7). While there is conflicting evidence for homeostatic-induced gene expression changes in tanycytes, there is consistent evidence between studies and species for seasonal/photoperiodic-induced changes in gene expression (Figure 2). In particular, tanycytes have been identified as key determinants of long-term seasonal changes in ingestive behavior and energy metabolism through their role in transport and regulation of thyroid hormone availability in the hypothalamus (8). The aim of this review is to summarize our current understanding of tanycyte biology and outline their key roles in nutrient and hormone sensing, and in directing neuroplasticity, and thereby regulating hypothalamic control of energy metabolism.

## Hypothalamic Tanycytes as Mediators of Energy Homeostasis

The blood–brain barrier (BBB) is a feature of the cerebral vasculature that restricts and regulates access of molecules to the brain, and therefore acts as a gatekeeper to the hypothalamic nuclei and beyond (9–11). However, despite the prominence of tanycytes within the ependymal layer of the third ventricle and their expression of a wide range of hormone receptors and nutrient sensors, their role in energy homeostasis is hotly debated. Early studies on tanycytes focused upon their barrier function (6). In response to food deprivation and a resulting fall in blood glucose, tanycytes undergo morphological changes and increase vascular permeability via enhanced secretion of VEGF-A (10). These reversible morphological alterations at the BBB suggest that nutritional state modulates the access of metabolic signals via tanycytes from the periphery to hypothalamic nuclei critical for energy homeostasis. However, the role of tanycytes as mediators of energy homeostasis extends beyond the morphological to the adaptive homeostatic and neuroendocrine.

In response to a fast, the hypothalamus–pituitary–thyroid (HPT) axis is downregulated through a reduction of thyrotropin-releasing hormone (TRH) synthesis in the paraventricular nucleus (PVN). The neurons of the PVN project to the median eminence and the terminals are in close proximity to the projections of β2 tanycytes. These cells express pyroglutamyl peptidase II (PPII), an ectopeptidase that hydrolyzes TRH, and thus controls the amount of TRH available to cause thyroid-stimulating hormone (TSH) synthesis and secretion in the anterior pituitary. *In situ* hybridization studies demonstrated that PPII and deiodinase 2 (DIO2) were increased in tanycytes following a fast (12). DIO2 removes an outer ring iodine atom, so converts the inactive form of thyroid hormone (thyroxine; T4) into the biologically active form triiodothyronine (T3). This is a common theme in tanycyte biology. Interestingly, increased DIO2 activity in tanycytes suppresses TRH secretion from the PVN via the local increase in T3 availability in the hypothalamus, and subsequent studies demonstrated that DIO2 in tanycytes is essential for regulation of the HPT axis (13–15).

An intriguing feature of tanycyte biology is that these cells are also activated by signals emanating from the adjacent *pars tuberalis* in the pituitary stalk. For example, TSH receptors located in tanycytes are activated by TSHβ produced in the *pars tuberalis*. This signal is transduced via both activation of adenylate cyclase and phosphorylation of extracellular signal-regulated kinases (ERK1/2), resulting in increases in DIO2 mRNA expression (16). In addition to fasting, overnutrition results in changes in tanycyte biology; ghrelin uptake/transport is attenuated following neonatal overfeeding (by reducing litter size) in the mouse (17). The lipopolysaccharide-induced cytokine upregulation of DIO2 expression in tanycytes and the stimulatory actions of pituitary adenylate cyclase-activating polypeptide both occur via increased intracellular cAMP and the NF-κB pathway (18, 19).

Interestingly, tanycytes express the insulin-independent glucose transporters GLUT1 and GLUT2, and also glucokinase. Indeed, in hypothalamic slice cultures, tanycytes respond to exogenously administered glucose, which stimulates Ca^2+^ ion fluxes and ATP release; effects that are then propagated across neighboring cells (20). This is further evidence that they function as nutrient sensors (21, 22). Furthermore, tanycytes express a number of enzymes involved in lipid metabolism, and monocarboxylate transporters, a family of transporters that mediate the facilitated diffusion of lactate, pyruvate, and ketone bodies. This suggests further possible mechanisms, whereby tanycytes mediate neuronal responses in the hypothalamus to changes in peripheral carbohydrate and fat metabolism (23, 24). Recently, a metabolic link between tanycytes and astrocytes, likely to impact hypothalamic lipid sensing, has been suggested (25). In addition, in leptin receptor deficient mice (*db/db*) and in mice treated with a leptin antagonist, leptin accumulates in the median eminence but fails to appear in the mediobasal hypothalamus, providing evidence that leptin’s signaling cascade begins in tanycytes in the median eminence, and then transitions to hypothalamic nuclei and neurons (26).

Further evidence supporting the neuroendocrine roles of tanycytes is provided by a series of experiments that targeted the fibroblast growth factor receptor 1 c isoform (FGFR1c). It was previously shown that antibody-mediated targeting of the FGFR1c receptor reduced body weight, adiposity, and insulin resistance in animal models of obesity and type II diabetes (27–29). Subsequent *in situ* hybridization studies in the Siberian hamster revealed a high level expression of the FGFR1c in tanycytes, consistent with previous qPCR studies in the mouse (30, 31). Targeting of the FGFR1c in the long day (LD) obese Siberian hamster peripherally and centrally via intracerebroventicular infusion of a monoclonal FGFR1c antibody reduced food intake and body weight, which was associated with a decrease in expression of DIO2 in the ependymal cell layer containing tanycytes (31). This further supports the hypothesis that tanycytes are an important component of the mechanism by which the hypothalamus integrates central and peripheral signals to regulate energy homeostasis. It also highlights a potential role in seasonal metabolic cycles, as the response to tanycyte manipulation was attenuated in short-day (SD) lean animals.

## Hypothalamic Tanycytes as Mediators of Seasonal Cycles

In response to seasonal changes in daylength, mammals such as the Siberian hamster and the F344 strain of photoperiodic rat undergo substantive behavioral and physiological adaptations, for example, in body composition, growth, and reproductive activity (32, 33). The retina is crucial to such adaptations; for example, optic nerve transection or bilateral enucleation prevents the synchronicity of seasonal reproduction (34, 35). Photoneuroendocrine pathways, where retinal information is conveyed to the suprachiasmatic nucleus, are well characterized, as is the neurochemical index provided by the secretion of melatonin by the pineal gland in response to changes in daylength (36). More recently, we have begun to appreciate the role and importance of the *pars tuberalis*, part of the pituitary stalk that contains a high density of melatonin receptors in all seasonal mammals and communicates to adjacent tanycytes in the hypothalamus (37). Emerging evidence suggests that tanycytes are an integral part of the mechanism that facilitates seasonal physiology and behavior in seasonal mammals. In addition to melatonin-regulated changes in secretion of paracrine factors including TSHβ and neuromedin U (NMU), this region undergoes structural changes in response to changing photoperiod, particularly in the thyrotrophs, which produce TSH (38–40). One consequence of this is that a significantly lower percentage of cells display exocytotic activity in SD, supporting the hypothesis that the *pars tuberalis* functions as an interface between photoperiodic stimuli and the endocrine system (41). Furthermore, the regulation of thyrotrophs is a melatonin-dependent process; pinealectomy blocks the SD-induced downregulation of TSHβ production, and treatment with melatonin can mimic the actions of SD (42, 43). As noted above, the TSHβ subunit has been shown to signal to tanycytes, and studies on the Syrian hamster, photoperiodic rat, and sheep have revealed that tanycytes express the TSH receptor, while local infusion of TSHβ into the third ventricle upregulates DIO2 in these glial cells (44, 45). It is of note that in juvenile photoperiodic rats, TSHβ also downregulates deiodinase 3 (DIO3) expression in the ependymal cell layer (44).

DIO3 is an enzyme in the tanycyte cell layer that opposes the action of DIO2, as it removes an inner ring iodine, and therefore deiodinates T4 into reverse T3, which is biologically inactive. Furthermore, it deiodinates T3 into the inactive metabolite di-iodothyronine (T2). In the adult Siberian hamster, rather than a LD-induced upregulation of DIO2 (Figure 2) that increases the local availability of T3, DIO3 is upregulated in response to SD (Figure 2), inactivating T3 or converting the precursor to T2 (32). This phenomenon is not limited to the Siberian hamster, it is also seen in male sheep exposed to SD for 14 weeks (46). It is predicted that the enhanced expression of DIO3 would have the same effect on local thyroid hormone availability in the hypothalamus as the downregulation of DIO2 observed in most other photoperiodic species (32). The biological significance of this predicted change in hypothalamic T3 concentrations was directly tested in the Siberian hamster by surgically inserting micro T3 implants into the hypothalamus, and exposing hamsters to changes in photoperiod. Such implants blocked the SD-induced weight loss and catabolism of fat depots and prevented SD-induced testicular regression (8). Correspondingly, T3-releasing implants stimulated appetite and induced body weight gain and reproductive recrudescence when placed in hamsters previously exposed to SD (47). The T3 microimplants blocked the SD-induced increase in VGF expression in the dorsomedial posterior arcuate nucleus, a potential regulator of seasonal changes in appetite and energy expenditure (8).

In addition to the clear effects of TSHβ derived from the *pars tuberalis* on deiodinase gene expression in tanycytes, other paracrine mechanisms may also be important in the regulation of deiodinases and tanycyte function. For example, ICV infusion of NMU decreases food intake and in obese mouse models increases physical activity, energy expenditure, and thermogenesis. Furthermore, NMU^−/−^ mice exhibit hyperphagia, increased body weight, and reduced energy expenditure. The actions of NMU are conferred by the NMU-2 receptor (48). Interestingly in photoperiodic rats in LD, NMU gene expression is upregulated in the *pars tuberalis*, while its receptor is upregulated in tanycytes (44, 49). It was subsequently shown that local infusion of NMU into the third ventricle of photoperiodic rats held in SD upregulated DIO2, thus mimicking the LD state (44). Similarly, the GPR50 receptor, which is homologous to the melatonin receptor MT1 but does not bind melatonin, is expressed in tanycytes (Figure 1) and has been implicated in adaptive thermogenesis and torpor (50). GPR50-null mice are resistant to diet-induced obesity; however, when fasted, they more readily enter a state of torpor. These effects appear to be mediated through TRH, as entry into torpor is reversed by treatment with TRH receptor agonists (51, 52). In the Siberian hamster exposed to SD, GPR50 expression is significantly reduced in tanycytes (Figure 2); this may contribute to bouts of adaptative thermogenesis, torpor, and more broadly energy balance (53). In response to SD, the thyroid hormone transporter monocarboxylate 8 (MCT8) is increased in tanycytes in the Siberian hamster, while fasting reversed this effect, further evidence supporting the role of thyroid hormone and tanycytes in the photoperiodic regulation of seasonal biology (54). Additionally, the thyroid hormone transporter organic anion transporter family member 1C1 (Oatp1c1) is photoperiodically regulated in tanycytes so potentially contributes to seasonal alterations in thyroid hormone transport [Figure 2; (55)]. Interestingly, the lactate (MCT2) and glutamate (GLAST) transporters, as well as glutamine synthetase, are reduced in tanycytes during SD (Figure 2), suggesting glutamate uptake and production of glutamine are diminished. Furthermore, glycogen phosphorylase and phosphofructokinase-C, rate-limiting steps in the metabolism of glycogen to glucose, are increased in tanycytes during SD [Figure 2; (56)].

Interestingly, T3 rapidly induces the RA-synthesizing enzyme retinaldehyde dehydrogenase 1 (RALDH1) in tanycytes (57). In photoperiodic rats, RALDH1 and -2 expression is reduced in SD, while the retinol transport protein stimulated by retinoic acid gene 6 homolog (STRA6) is reduced by SD (58, 59). Furthermore, expression of transthyretin (TTR), a common transporter for vitamin A and its metabolite retinoic acid, is downregulated under SD in the tanycytes of photoperiodic rats, while cellular retinoic acid binding protein (CRBP1), a retinoic acid transport protein, is downregulated in SD photoperiods in tanycytes in Siberian hamsters. The latter effects are reversed by pinealectomy, which suggests that the mechanism is dependent upon melatonin (53). Furthermore, cellular retinoic acid binding protein-2 (CRABP-2) and members of the nuclear retinoic acid receptor and retinoid X receptor families are reduced in response to SD in the Siberian hamster (53, 60). Interestingly, retinoic acid regulates the ability of tanycytes to proliferate and generate new cells in the hypothalamus highlighting another possible role for tanycytes (5).

## Hypothalamic Tanycytes as a Stem Cell Niche

A number of studies support the existence of hypothalamic stem cells capable of generating new neurons in a variety of species. However, the location and identity are hotly disputed. Recent *in vitro* and *in vivo* studies have suggested that they are located within the mediobasal hypothalamus parenchyma and could represente NG2-expressing oligodendrocyte progenitor cells (61, 62). Contrasting studies have suggested that subpopulations of tanycytes constitute the source (63–66). This in itself, however, is controversial as both α- and β-tanycytes have been identified as the possible neurogenic niche, as well as a possible role for insulin-like growth factor (63, 65, 67). Interestingly, in one study, exposure of mice to a high fat diet depleted numbers of putative hypothalamic stem cells, which was associated with impaired glucose tolerance and subsequent obesity (68). However, rather contradictory results were reported in the study that demonstrated increased numbers of cells labeled with the thymidine analog BrdU in the hypothalamic ventricular zone in mice maintained on a high fat diet (64). Furthermore, in the latter study, focused irradiation of the hypothalamus inhibited cell division that was associated with reduced body weight gain on a high fat diet, suggesting that new cells produced in the hypothalamus might have an anabolic function (64). More recently, increased ciliary neurotrophic factor signaling was detected in tanycytes close to the median eminence in obese mice on high fat diet, further supporting the hypothesis that positive energy balance is associated with enhanced hypothalamic neurogenesis (69).

In addition to high fat diet, photoperiodic stimuli regulates cell division in the adult hypothalamus. Exposure to SD increased vimentin labeling in hypothalamic tanycytes of sheep and increased numbers of BrdU-positive cells in the sheep hypothalamus, though a substantive proportion of these expressed a microglia marker so were not destined to become neuronal (70, 71). Following the transition from LD to SD, an increase in cellular proliferation is apparent in the hypothalamus of Syrian hamsters; in the Siberian hamster, the intermediate filament protein, and neural stem cell marker nestin is downregulated during SD (53, 72). Further studies are clearly required to determine whether the reported seasonal changes in BrdU uptake or expression of cell cycle markers such as Ki67 truly reflect altered neurogenesis, or whether new cells integrate into functional circuits in the hypothalamus. However, given the evidence above regarding photoperiod-induced changes in thyroid hormone availability in the hypothalamus, and the extensive evidence that the thyroid hormone system is implicated in neural division and differentiation, it seems very likely that plasticity of cell division and connectivity in the hypothalamus will be identified as a core feature of seasonal cycles (73, 74). Finally, it has been observed that the ability of tanycytes to proliferate postnatally declines with age: incorporation of the S-phase marker BrdU in β-tanycytes deteriorates between P7 and P45, while no incorporation is seen by 12 months of age (63). Furthermore, tanycyte numbers declines by almost 30% with increasing age as well as inducing significant morphological and anatomical changes; processes become thicker and disorganized in the pericapillary zone, with a loss of perpendicular orientation (75). This poses further tantalizing questions regards their metabolic role in relation to aging, and whether seasonal cycles might be considered as arrested or even reversible aging.

## Conclusion

Identifying the mechanisms by which mammals naturally regulate appetite and body composition across the year should provide insights into how long-term improvements in metabolic health could be promoted in man. Tanycytes are the only cell type in the hypothalamus that shows major changes in gene expression across a seasonal cycle, so are a likely regulator of long-term changes in energy balance. Tanycytes have a privileged position as a nutrient and hormone sensor with projections to the metabolic brain, and potentially function as a neural stem cell niche, highlighting a number of mechanisms that could influence energy intake and expenditure in the long term. Experimental studies in the hamster have already confirmed that changes in thyroid hormone processing by tanycytes are part of this seasonal programming of the hypothalamus.

## Author Contributions

JL and FE drafted and revised the manuscript.

## Conflict of Interest Statement

The authors declare that the research was conducted in the absence of any commercial or financial relationships that could be construed as a potential conflict of interest.
